# ﻿Taxonomic note of *Parnassia* (Celastraceae): the identity of *P.yui*

**DOI:** 10.3897/phytokeys.251.140807

**Published:** 2025-01-29

**Authors:** Shi-Qi Wang, Xue-Mei Zhang, Yu-Min Shu

**Affiliations:** 1 College of Life Science, China West Normal University, Nanchong 637002, China China West Normal University Nanchong China; 2 Key Laboratory of Southwest China Wildlife Resource Conservation (Ministry of Education), Nanchong 637002, China Key Laboratory of Southwest China Wildlife Resource Conservation (Ministry of Education) Nanchong China

**Keywords:** Morphology, *Parnassia*, staminode, synonymy, taxonomic uncertainty

## Abstract

*Parnassiafarreri* is characterised by the petals with long-ciliate petal claw and the broad spatulate staminodes with shallow crenations distally. A sympatric and poorly-known taxon, *P.yui*, is recorded only from the type collection that morphologically similar to *P.farreri*. Based on field investigations, examination of herbarium specimens (including the types) and analysis of protologues and distributions, *P.yui* is hereby reduced to a synonym of *P.farreri.* Field and anatomy photographs and an updated morphological description of *P.farreri* are provided. *Parnassiafarreri* is preliminarily designated as Least Concern according to the IUCN Red List guidelines.

## ﻿Introduction

*Parnassia* L. (Linnaeus 1753) is a fairly homogeneous and distinguishable genus of Celastraceae (APG IV 2016; Ball 2016), consisting of small and glabrous perennial herbs, with a solitary, bisexual and pentamerous flower that has five staminodes borne on an unbranched scape (Ku 1987; Ku and Hultgård 2001). Species of *Parnassia* predominantly occur in alpine and arctic regions of the Northern Hemisphere, which most diverse in Pan-Himalaya and southwest China (Phillips 1982; Wu et al. 2003; Simmons 2004; Wu 2005). The most recent checklist of *Parnassia* worldwide was accomplished by Shu et al. (2017) and contains 61 species, two subspecies, 11 varieties and one form. Since then, more than ten names have been reduced to synonyms (Shu et Zhang 2017; Wang et al. 2018; Yu et al. 2018; Ma et al. 2020; Dai et al. 2021) and two new species were described (Zhang et al. 2019).

**Figure 1.**
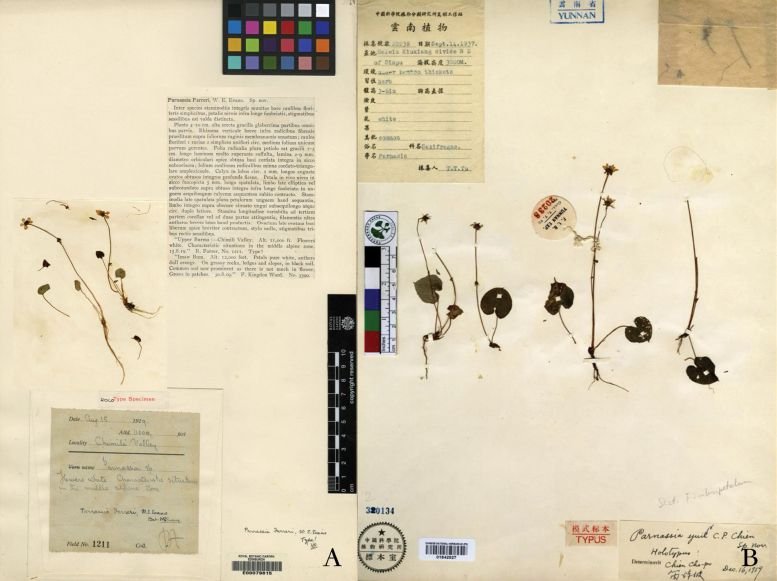
Holotypes of *Parnassiafarreri* (**A** E00079815) and *P.yui* (**B** PE01842927).

*Parnassiafarreri* W. E. Evans (1921: 174) was formally described, based on the specimens of *R. Farrer 1211* (Fig. 1A) which was collected from Myanmar. The petal base of this species is abruptly contracted into a claw with long ciliated margin, that is distinctive in this genus. *Parnassiayui*Jien (1963: 256), another species with similar morphological features and neighbouring distribution to *P.farreri*, was described, based on a single collection (T. T. Yu 20238, Fig. 1B, 24 individuals mounted on four sheets) from Yunnan Province, China. According to the original description, the morphological differences between *P.yui* and *P.farreri* include the larger leaves blades (12–24 × 12–24 mm vs. 2–14 × 2–14 mm), the acute or acuminate (vs. rounded) apexes of narrower petals and the three lobed (vs. entire or obscurely sinuate) apex of staminodes.

Through observation of herbarium specimens, we found these two species are extremely similar in morphology and there is obvious overlap in the shape of leaf blades and staminodes. Therefore, the aim of this study was to clarify the classification relationship between *P.farreri* and *P.yui* by re-evaluating their morphological characteristics.

## ﻿Materials and methods

The type specimens of *P.farreri* and *P.yui*, along with other related specimens deposited at KUN, PE and SITC, were meticulously inspected. Additionally, digitised material sourced from Herbaria CAS and E (acronyms according to Thiers (2024)) were carefully examined. Field investigations were carried out during September to October of 2024 in Gongshan County, Yunnan Province, China. Eleven individuals with well-preserved leaves and flowers from the type specimens of *P.yui* and fifty individuals of *P.farreri* from the wild were used to conduct the morphological measurement and comparison. Eight morphological characters, i.e. leaf length, leaf width, ratio of leaf length/width, petal length, petal width, ratio of petal length/width, number of staminode lobes and depth of staminode lobes were measured in this study. Principal Component Analysis (PCA) was performed to investigate the morphological variations between *P.farreri* and *P.yui*.

## ﻿Results

**Figure 2.**
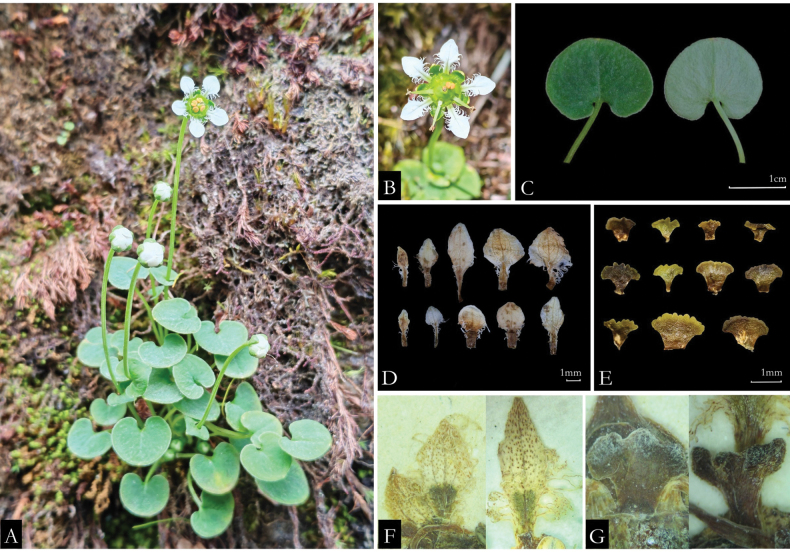
Morphology of *Parnassiafarreri***A** habitat **B** flower **C** leaves **D** variation of petals **E** variation of staminodes and *P.yui***F** petals **G** staminodes, photographed from *T. T. Yu 20238*, the type specimen.

**Figure 3.**
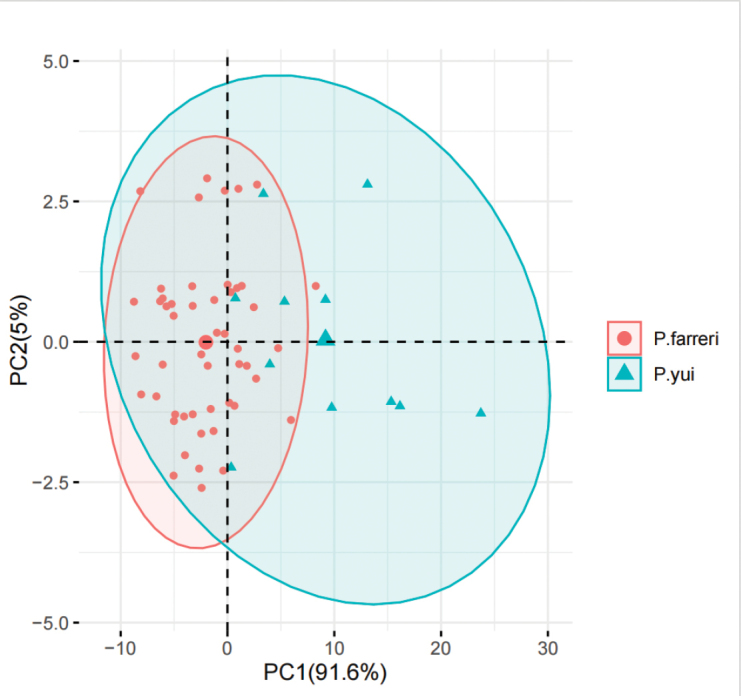
The Principal Component Analysis (PCA) plot for the morphological variations between *Parnassiafarreri* and *P.yui*. The confidence ellipse level is 95%.

During the specific field survey of *P.farreri* populations in southwest China, we documented continuous variations of leaf blades, the shapes of petals and staminodes (Fig. 2A–E), even sometimes within individuals. No specimens of *P.yui* were traced, except the type materials. Amongst the individuals from the type materials of *P.yui*, only a few have oblanceolate petals and shallowly 3-lobed staminodes, that match the original description. Additionally, most individuals have triangular-ovate to ovate-spartulate petals, as well as irregular dentate staminodes apexes (Fig. 2F–G). Principal Component Analysis (PCA) could not distinguish these two taxa, the 95% confidence ellipse of *P.farreri* being almost included in that of *P.yui* (Fig. 3). Furthermore, the distribution of *P.yui* is limited in Gongshan County, Yunnan, China and, in this region, more than ten specimens of *P.farreri* have been collected. The latter taxon is also distributed in Myanmar and India (Shu et al. 2017; Pankaj et al. 2018). Since the lack of clear morphologic distinctions and the overlapping distribution range, we hereby propose to reduce *P.yui* to a synonym of *P.farreri*.

### ﻿Taxonomic treatments

#### ﻿ Parnassia farreri

Taxon classificationPlantaeCelastralesCelastraceae

W.E. Evans (1921: 174)

3A761928-6EDC-520E-9BD0-4D61E050175B

##### Type.

Myanmar • Chimely Valley, in the middle alpine zone, 3300 m, 15 August 1919, *R. Farrer 1211* (holotype: E00079815 photo!).

#### ﻿ Parnassia yui

Taxon classificationPlantaeCelastralesCelastraceae

Jien (1963: 256)

653AA851-0874-5EF0-84B5-E66806ACB5AA

##### Type.

China • Yunnan, Salwin-Kiukiang Divide, northeast of Sinpu, under bamboo thickets, 3000 m, 14 September 1938, *T. T. Yu 20238* (holotype: PE01842927!; isotypes: PE01842926!, KUN1205235!, KUN1205238!)

##### Description.

Perennial herbs, glabrous. Rhizome sympodial. Stems 1 to 4, slender, 3–16 cm, with a tiny cauline leaf near middle. Basal leaves 1 to 7, petiole 1–4.5 cm; blade reniform, broad cordate or orbicular, 0.2–2.4 × 0.2–2.8 cm, base cordate, apex rounded or apiculate. Flower 0.8–1.5 cm in diam.; hypanthium turbinate. Sepals oblong, ovate or lanceolate, 2–3 × 1–2 mm, margin entire, apex acute. Petals white, densely purple-brown punctuate when dried, oblanceolate, triangular-ovate, to ovate-spartulate, 3–6 × 1.5–3.5 mm, base contracted into a claw ca. 2 mm, margin long fimbriate proximally, entire distally, apex acute to rounded-obtuse. Anthers ellipsoid, filaments 2–2.5 mm. Staminodes flat, broadly spatulate, 2 × 1–2 mm, apex shallowly 3-lobed, dentate, undulate or rounded. Ovary superior, broadly ovoid; style ca. 1 mm; stigma 3-lobed. Capsule depressed ovoid. Seeds brown, glossy, oblong.

##### Phenology.

Flowering from July to September, fruiting from August to October.

##### Distribution.

China, Myanmar and India.

##### Habitats.

Under shrubs, grassy rocks, ledges and slopes at an elevation of 3000–3900 m.

##### Additional specimens examined.

**China. Yunnan, Gongshan County**: • 3252 m alt., 21 August 2024, *Y.M. Shu et. al. s1166* (SITC); • 3336 m, 21 August 2024, *Y.M. Shu et. al. s1168* (SITC); • 3399 m alt., 21 August 2024, *Y.M. Shu et. al. s1170* (SITC); 30 July 2013, *X.H. Jin et al. st1518* (PE01979311!); • 3600 m alt., 29 July 2013, *X.H. Jin et al.* st1357 (PE!); • 3300 m alt., 22 July 2013, *X.H. Jin et al. ST0741* (PE!); 2 September 2011, *S.X. Yu et al. 6521* (PE!); 24 August 2009, *WWZ 126* (KUN!); • 3250 m alt., 19 September 2003, *J.H. Chen 3051* (KUN!); • 3600 m, 19 September 2003, *J.H. Chen 3053* (KUN!); 19 August 2003, *J.M. Lu et al. 2319* (KUN!); • 3080 m alt., 2 October 2002, *Gaoligong Shan Biodiversity Survey16895* (CAS0321223!); • 3400 m alt., 9 September 1940, *K.M. Feng 7619* (PE00866103!, KUN0437239!, KUN0437240!); • 3300 m alt., 20 August 1937, *T.T. Yu 22447* (PE01982464!, PE01982465!, PE00866102!); • 3100 m alt., 1 October 1935, *Q.W. Wang 67226* (PE00866104!); **Fugong County**: • 3700 m alt., 16 August 2005, *H. Li et al. 28515* (CAS0321224!); • 20 August 2003, 2800 m alt., *J.M. Lu et L.M. Gao 2327* (KUN!); **Lushui County**: • 3050 m alt., 08 August 2011, *X.H. Jin et al. 11171* (PE02025103!).

**Myanmar.** • 3600 m, 30 August 1919, *F. Kingdon Ward. 3599* (E00275515 photo!).

##### Conservation status.

The conservation status of both *P.farreri* and *P.yui* have not been evaluated before. At present, *P.farreri* has been reported from China, Myanmar and India. Based on our field investigations, numerous individuals could be easily discovered under shrubs, in grassy slopes or on ledges, indicating the population survives and regenerates well. Thus, we propose to list *P.farreri* as Least Concern (LC) according to the IUCN Red List Categories and Criteria (IUCN Standards and Petitions Committee 2022).

## Supplementary Material

XML Treatment for Parnassia farreri

XML Treatment for Parnassia yui

## ﻿Additional information

### Data availability

All of the data that support the findings of this study are available in the main text.
